# Evaluation of Peptide/Protein Self-Assembly and Aggregation by Spectroscopic Methods

**DOI:** 10.3390/molecules25204854

**Published:** 2020-10-21

**Authors:** María Florencia Pignataro, María Georgina Herrera, Verónica Isabel Dodero

**Affiliations:** 1 Department of Physiology and Molecular and Cellular Biology, Institute of Biosciences, Biotechnology and Translational Biology (iB3), Faculty of Exact and Natural Sciences, University of Buenos Aires, Buenos Aires C1428EG, Argentina; mfp@qb.ffyb.uba.ar; 2 Institute of Biological Chemistry and Physical Chemistry, Dr. Alejandro Paladini, University of Buenos Aires-CONICET, Buenos Aires C1113AAD, Argentina; 3 Organic and Bioorganic Chemistry, Department of Chemistry, Bielefeld University, 33615 Bielefeld, Germany

**Keywords:** proteins, peptides, self-assembly, dyes, aggregation, fibrils, spectroscopy

## Abstract

The self-assembly of proteins is an essential process for a variety of cellular functions including cell respiration, mobility and division. On the other hand, protein or peptide misfolding and aggregation is related to the development of Parkinson’s disease and Alzheimer’s disease, among other aggregopathies. As a consequence, significant research efforts are directed towards the understanding of this process. In this review, we are focused on the use of UV-Visible Absorption Spectroscopy, Fluorescence Spectroscopy and Circular Dichroism to evaluate the self-organization of proteins and peptides in solution. These spectroscopic techniques are commonly available in most chemistry and biochemistry research laboratories, and together they are a powerful approach for initial as well as routine evaluation of protein and peptide self-assembly and aggregation under different environmental stimulus. Furthermore, these spectroscopic techniques are even suitable for studying complex systems like those in the food industry or pharmaceutical formulations, providing an overall idea of the folding, self-assembly, and aggregation processes, which is challenging to obtain with high-resolution methods. Here, we compiled and discussed selected examples, together with our results and those that helped us better to understand the process of protein and peptide aggregation. We put particular emphasis on the basic description of the methods as well as on the experimental considerations needed to obtain meaningful information, to help those who are just getting into this exciting area of research. Moreover, this review is particularly useful to those out of the field who would like to improve reproducibility in their cellular and biomedical experiments, especially while working with peptide and protein systems as an external stimulus. Our final aim is to show the power of these low-resolution techniques to improve our understanding of the self-assembly of peptides and proteins and translate this fundamental knowledge in biomedical research or food applications.

## 1. Setting the Frame and Initial Considerations

Protein self-assembly plays a pivotal role in cellular physiology. It is a necessary process for the formation of any ordered protein structure [1]. Amelogenin proteins, which direct dental mineralization [2], are a remarkable example of self-assembled proteins that are found in structural tissues. On the other hand, self-organization and aggregation of proteins also takes part in physiopathological processes, as it is observed in different neurodegenerative diseases, where amyloid fibrils are deposited on different parts of the central nervous system [3]. The formation of protein aggregates has also become a fascinating research area, not only for mitigating diseases such as Alzheimer’s and Parkinson’s, but also in the pharmaceutical and food industries. In the pharmaceutical context, the uses of human recombinant proteins to treat a wide variety of diseases such as cancer, Hemophilia A and multiple sclerosis established the necessity to control and study the aggregation tendency of these formulations to avoid efficiency reduction and exacerbate immune response [4,5]. In this respect, a meticulous analysis of the therapeutic protein is needed in terms of protein structure, stability and oligomeric state. Moreover, in the food industry, the aggregation processes of whey proteins from milk [6], wheat gliadin and glutenin [7], and peanut proteins [8] are some examples that are continuously studied. The formed aggregates affect not only the organoleptic properties of the final product, but also its digestibility, bioavailability of amino acids and, in some cases, they can even induce or reduce inadequate immune responses to a toxic protein [9,10].

On the other hand, the aggregation capability of proteins from natural sources is used to generate nanoparticles for drug-delivery as in the case of whey, zeins and gliadins [11,12,13]. In this context, the use of techniques that allow the analysis of protein solutions to determine the presence of aggregates in a fast, reproducible and qualitative way is of key importance.

Nowadays, there is a wide range of possible methods for assessing protein and peptide self-assembly phenomena. However, to obtain molecular information, the use of specialized core facilities and also specific training on them is required. In this sense, spectroscopic methods like UV-Vis, fluorescence and circular dichroism together make a simple approach for evaluating proteins and peptides and assessing their self-assembly processes. In this regard, these spectroscopic techniques provide unique information in the case of complex systems, such as those composed by a mixture of proteins, where their analysis by sophisticated techniques as NMR (Nuclear Magnetic Resonance) is not easy to implement. The simplicity of these techniques in terms of implementation and analysis and their availability in multidisciplinary laboratories make them the first choice in the evaluation of protein and peptide self-assembly. Because of these reasons, we chose them as the topic of this review. It is worthy of mentioning that NMR is one of the most powerful tools for providing information about protein structure, dynamic and self-association at the atomic level, and is outside the scope of this review. The system needs to be labelled with ^15^N and ^13^C, and the broadening of the spectra peaks occurs when the size of the protein is above 30 kDa. As a consequence, methodologies such as Transverse relaxation-optimized spectroscopy (TROSY), methyl-TROSY and Cross-correlated relaxation-enhanced polarization transfer (CRINEPT) [14,15] have been developed to analyze self-assembled systems with a molecular mass above 30 kDa. Another NMR approach for studying supramolecular systems is solid-state nuclear magnetic resonance (ssNMR), which provides structural and dynamical information of complex biological systems especially in the case of protein aggregates and fibrils, such as the ones observed in amyloids [16,17,18]. For a deeper understanding of this technique, we suggest assessing the works cited here and some more specialized bibliography [19,20].

Moreover, spectroscopic methods are generally complemented with imaging tools such as Cryo-Transmission, Transmission and Scanning Electron microscopies, as well as Atomic Force and fluorescence microscopies, thus obtaining information about the morphology and topology of the systems [21]. The description of these valuable methods is not the focus of this review, but considering their relevance in the visualization of self-assembly systems, we encourage the reader to refer to these well-written reviews and books [21,22,23,24,25].

### An Overview of Protein Structure and Self-Assembly

Proteins and peptides develop a variety of activities in the cell. These molecules adopt a local folding referred to as the secondary structure (i.e., α-helix or β-sheet structure) and a tridimensional location of the secondary structure in space, known as the tertiary structure. Moreover, some proteins adopt a quaternary structure, which implicates the association between protein subunits and their arrangements from dimers to oligomers. [26,27]. The self-association of proteins in different oligomeric states is mainly governed by nonbonded interactions such as the Van der Waals forces, hydrogen and ionic bonds and π–π interactions [28]. However, in some cases, the formation of specific disulfide bonds is critical for protein oligomerization [26]. The size of the self-associated superstructures could range from nm to µm. Some systems are stabilized in the solution, forming a dispersion; meanwhile, others form insoluble amorphous aggregates or ordered ones, like fibrils [29].

Amorphous aggregates as spherules and fractal-like clusters can be detected in the first stages of the self-assembly process, as in the case of the 33-mer gliadin peptide related to celiac disease [30] or the silk protein sericin [31]. In the case of fibrils, they are classified as non-amyloid and amyloids. Representative non-amyloid fibrillar structures are mainly related to motility and scaffold functions in the cell, such as actin fibrils, microtubules, collagen, among others [32]. On the other hand, amyloid fibrils are well known to be a hallmark of neurodegenerative diseases such as Alzheimer’s, Parkinson’s, and type II diabetes, among other pathologies [3].

**Figure 1:**
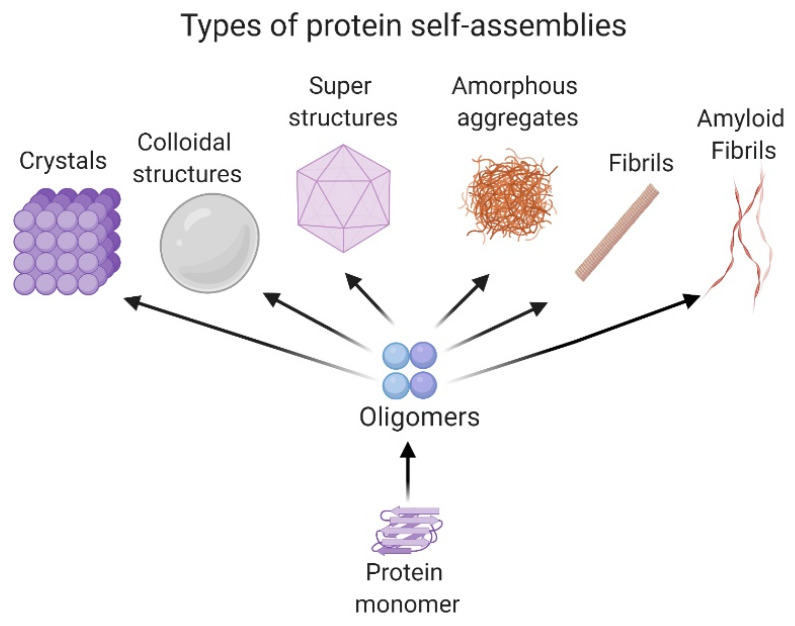
Schematic representation of the different types of the most observed protein self-assemblies. Image created with Biorender.com.

Recently, it was demonstrated that colloidal protein behavior has an essential role in self-assembly processes, as occurs in condensation and Ostwald ripening [33,34]. Ostwald ripening explains the formation of protein droplets in a liquid system, such as the nucleolus or the cytoplasm, and is of great importance in cellular physiology and stress response [35]. Interestingly, there are self-associated proteins that form regular spatial patterns. One of the most well-known is the formation of superstructures like viral capsids, which require a specific number of monomers to generate the self-associated system. In particular, conditions of salts, temperature and pH, proteins could also associate to form crystals. This phenomenon is of particular interest because it allows the 3D structure resolution of the protein by X-ray diffraction [36]. All the structures presented are shown in Figure 1.

Secondary structure conversion of peptides and proteins from native conformation towards a β-sheet structure, independently of the protein sequence, has been described in a variety of proteins and especially in the amyloidogenic ones. In this case, non-branched fibrils are formed with a β-sheet conformation named cross-β, where the hydrogen bonding direction is parallel to the fiber axis, and the β-strands are perpendicular, like the rungs of a ladder [37]. Recently, it was pointed out that the α-sheet structure, referred to as “polar pleated sheet”, has an essential role in the formation of toxic oligomers. This structure is highly similar to the β-sheet, except that the carbonyl oxygen atoms are aligned on one face of a strand and the NH groups on the other, instead of alternating with each other, giving rise to different physical properties to the protein. This structure was found in the early stages of the Aβ-peptide [38,39] and the transthyretin protein (TTR) aggregation [40].

Moreover, another relevant motif for protein self-assembly is Polyproline II (PPII). This secondary structure is a left-handed helix that does not depend on the formation of hydrogen bonds in the backbone or salt bridges, but regularly establishes hydrogen bonds with the solvent [41]. This structure has been shown to be highly abundant, especially in structural proteins like collagen and exposed protein segments [42,43]. Additionally, it can interconvert into other forms such as β-turns and β-strands because of the proximity of the corresponding dihedral angles [44]. PPII plays a vital role in protein–protein interactions [45] and aggregation, as it was detected as the intermediate motif during the self-aggregation of lysozyme [46], the Aβ-peptide [47] and gliadin peptides [48,49].

## 2. UV-Vis Absorption Spectroscopy and Turbidity Are Initial Approaches for Detecting the Self-Assembly/Aggregation-Prone Tendency of Proteins

**Figure 2:**
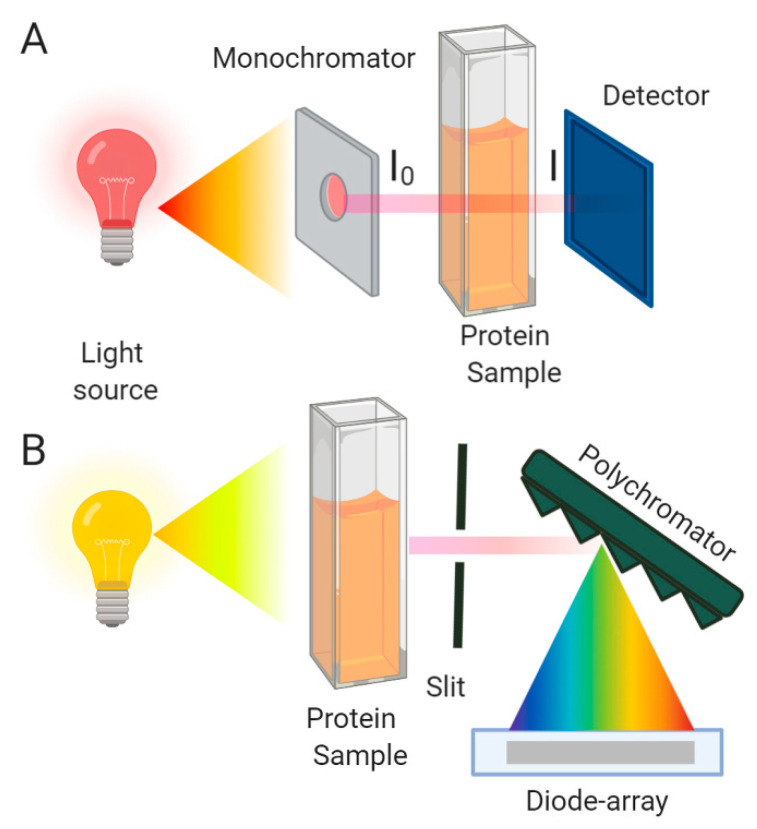
Schematic representation of the process of absorbance and the instrumentation used. Diagrams of (**A**) conventional and (**B**) photodiode array spectrophotometers. The image was created with Biorender.com.

UV-Vis spectroscopy is a fast, non-destructive technique that requires a relatively small amount of protein sample for the analysis. It is a commonly used method available in most laboratories due to its versatility and utility [50]. In recent decades, it has been improved by the invention of diode-array detectors, which nowadays are one of the most used detectors in spectrophotometers (Figure 2). This detector, in combination with new spectral analysis methods, has renewed UV-Vis applications, especially for accessing protein conformational changes and aggregation [51]. There are direct and indirect approaches for determining protein aggregation. The direct one is the most used and consists of the straight detection of aggregates in solution; meanwhile, the other method evaluates protein structural changes upon self-assembly.

### 2.1. Principles and General Considerations

UV-Visible spectroscopy is based on the absorption of light from a chromophore due to the transition of electrons from the ground to an excited state. Spectra are obtained by acquiring the absorption of light at different wavelengths or frequencies. The general Equation (1) is

(1) $$A_{\lambda }=log_{10}\left(I_{0}/I\right)=kl$$

where *k* = *εc*, $A_{\lambda }$ is the absorbance at a specific wavelength, $I_{0}$ is the intensity of the incident light, I is the intensity of the light that emerged from the sample. k is a constant that is proportional to the chromophore’s concentration, and its molar extinction coefficient (ε) over a 1 cm path length (M^−1^/cm) and l is the optical pass of the sample (Figure 2). The direct linear relationship between the absorbance and concentration is known as the Lambert-Beer law.

A conventional laboratory spectrophotometer is presented in Figure 2A. It is composed by deuterium (190–400 nm) or/and a tungsten lamp (400–2500 nm) as the sources of light. A monochromator generally selects the emission wavelength. The light that comes out from this dispositive (I_0_) has a narrow band of wavelengths and is directed to the cuvette containing the sample. The emerging light from the cuvette (I) goes directly to the detector, that generally consists of a photomultiplier or a photodiode. Each one presents advantages and disadvantages, and the silicon photodiode arrangement is one of the most used due to its lower cost and higher versatility. Moreover, the photodiode array is a solid-state device and is more secure and reliable than a photomultiplier tube [52]. It should be noted that some instruments with photodiode arrays may contain a slightly different scheme from that shown in Figure 2B, where the entire spectrum of light is transmitted through the sample and passed into an entrance slit of a polychromator. The polychromator is an enhanced monochromator, where the wavelength scanning is accomplished by electronic scanning of the multichannel detector. This multichannel dispositive can detect as many wavelengths simultaneously as their number of individual diodes or pixels [52,53].

Generally, when collecting a spectrum, there are some technical aspects to take into account. At first, it is necessary to select the right cuvette and buffer for the experiment. The cuvettes could present different geometries; the most used are the cylindrical or rectangular ones, but the size and shape depend on the characteristics of the spectrometer and the sample to analyze. Additionally, the cuvette material is an important parameter to consider. If collecting a spectrum in the visible range, the material could be optical crystal or plastic (380–780 nm), but if the analysis would take place in the UV-region, a quartz cuvette is needed (190–300 nm). In the case of the sample buffer, it is better to select one that does not absorb in the region of the light spectrum of interest. For example, imidazole buffers should be avoided in protein samples due to imidazole absorbance near the protein region. Additionally, the corresponding spectrum of the working buffer might be collected before this for the specimen to detect possible interferences and then subtracted to the sample spectrum before the data analysis.

### 2.2. Indirect Methods: Evaluating Protein Absorption Spectra

#### 2.2.1. Intrinsic Chromophores

The absorption of light by the protein chromophores allows indirect determination of the protein structure changes upon aggregation. In proteins, the amide bond absorbs in the far UV region (180–230 nm), where two significant transitions occur, one at 195 nm (π → π ∗) and a second one weaker at 220 nm (*n* → π ∗). In terms of secondary structure, the α-helix, β-sheet and random coil exhibit a different UV-spectra in this region; however, its implementation to distinguish between secondary structures is limited. From one side, there is high over-lapping of signal and the fact that other substances like oxygen and other inorganic molecules absorb in this region.

The Near-UV-region (240–295 nm) is where the lateral chains of the aromatic residues such as tyrosine, tryptophan, phenylalanine and cysteine absorb. The aromatic ones absorb due to π → π ∗ transitions, and the contribution of each amino acid is different. The indole group from tryptophan presents a maximum near ~280 nm and a less intense transition around ~292 nm, usually observed as a shoulder form of the spectra. Tyrosine absorbance is lower than Trp with a maximum seen at ~276 nm and two small shoulders at ~267 and 280 nm. Phenylalanine exhibits the weakest transition around 250 to 270 nm region, appearing as multiple subtle inflexion points, with a peak centered near 260 nm.

As a consequence, the Near-UV spectrum of a protein is dominated by the contributions of tyrosine and tryptophan. The aromatic amino acids do not absorb above 310 nm, and the protein absorbance should be near zero above this wavelength. Due to the high ε of tyrosine and tryptophan at 280 nm, the absorbance at this wavelength is used for determining the concentration of a pure protein sample, when the primary sequence is known. In Table 1, the characteristics of each aromatic amino acid and the equation to calculate the ε at 280 nm are presented in detail, which can be calculated by the online tool ProtParam from ExPASy [54]. For the mentioned characteristics, the 280–295 nm range is the preferred one to follow protein structural changes and unfolding [55].

On this subject, it is essential to mention that the aromatic residues could suffer from oxidation and ionization reactions that could induce a change of the absorbance spectrum or the formation of a new band. One of the most characteristic ones is the oxidation of tyrosine to di-tyrosine, which results in the appearance of a new peak near ~320 nm [50,55]. This new bond is characteristic during protein crosslinking and a hallmark of protein aggregation in the case of insulin after UV-exposure [56].

**Figure 3:**
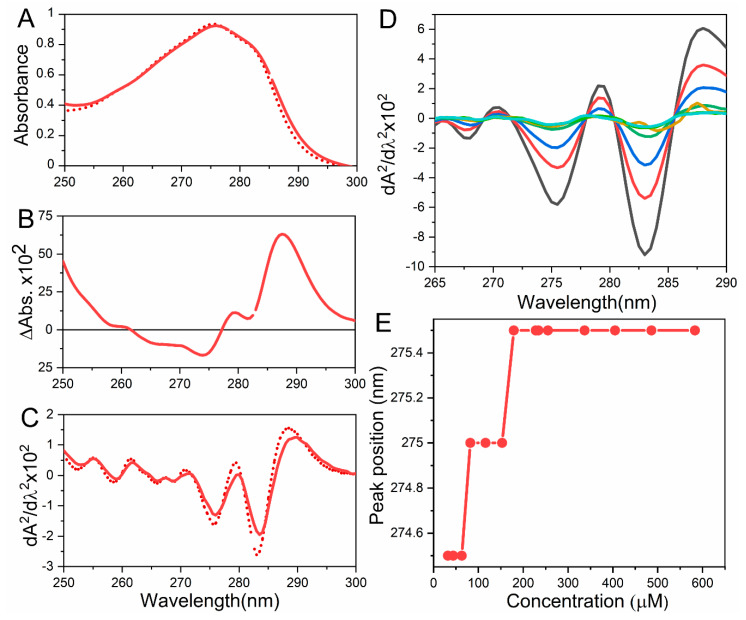
(**A**) Simulated UV Absorption Spectra of a model peptide containing phenylalanine and tyrosine that oligomerizes. (**B**) Difference spectrum between the native (solid line) and unfolded (dotted line) form. (**C**) Second derivative spectra of the two spectra showed in (**A**). (**D**) Experimental second derivative spectra obtained during 33-mer gliadin peptide self-assembly into soluble aggregates in a concentration-dependent manner. The peptide possesses tyrosine and phenylalanine in its sequence. (**E**) Representation of the changes in the minimum of the second derivative at 274–275 nm. A redshift was observed upon titration with increasing concentrations of 33-mer which is related to the self-assembly process. The critical aggregation concentration obtained was above 80 µM. Figure (**D**,**E**) were adapted from Herrera et al. [30].

Notably, the maximum absorption of tryptophan and tyrosine is sensitive towards the microenvironment. Therefore, a peak displacement to lower wavelengths (i.e., blueshifts) is indicative of exposure to a more polar solvent environment. In contrast, shifts to longer wavelengths (i.e., redshifts) imply that these residues are located in a more buried environment and less exposure to the solvent. These could be quickly evaluated by analyzing the mathematical difference between both spectra (Figure 3). In Figure 3A, the spectra of a peptide with tyrosine and phenylalanine, which forms oligomers at 25 °C (solid line), and its unfolded state (dot line) are presented. The difference spectrum between the native and unfolded is shown in Figure 3B, showing that the main difference occurs at 285–291 nm, which corresponds to tyrosine exposure to the solvent. In the case of tryptophan-containing proteins, a shoulder around 290–300 nm (blue shifted, to lower wavelengths) is commonly observed when the residue goes from non-polar to polar environments. A useful tool to follow conformational alterations is the second derivative of spectra, which makes it possible to track shifts on the positions of the peaks accurately. The intensity of the second derivative is employed to quantify protein and determine tyrosine/ tryptophan ratios because of it is not perturbed by the scattering of the light due to the presence of protein aggregates [51]. In Figure 3C, the second derivative spectrum of A is presented, showing that there are two prominent peaks in the specimen. In the native state (*solid lines*) one of them is at 275.9 nm and the other at 283.5 nm. After unfolding (*dashed line*), there is a blueshift to 275.4 nm and 283 nm, respectively [51,57]. The second derivative is commonly employed to evaluate conformational changes of proteins associated with self-assembly. This approach was used in the evaluation of the self-assembly of the 33-mer peptide at different concentrations at it is observed in Figure 3D, E. Here, a red-shift is observed indicating that the tyrosines of the peptide are located in a more hydrophobic environment upon aggregation [30]. Additionally, this approach was used to evaluate the aggregation upon cation binding of Azurin and RNAse T1 [58], human serum albumin aggregation into fibrils [59], gliadin self-organization in aqueous solutions [60] and the assembly of the Ac-Phe-Phe-Cys-NH_2_ (Ac-FFC-NH_2_) amyloid peptide model [61].

#### 2.2.2. Extrinsic Chromophores

Notably, there are some proteins with co-factors such as NADH, FADH and *heme* groups in their composition that could absorb light, generally in the Near-UV and Visible part of the light spectrum [62]. In the case of the *heme* group, it presents a typical absorption in the range of 350–700 nm, with the maximum of absorption generally around 412 nm, referred to as the Soret band. Meanwhile, the bands between 450–700 nm are named as Q bands. The changes in the absorption of the Soret band are widely employed to evaluate the aggregation of alpha-1-microglobulin upon *heme* binding [63] and haemoglobin self-association in the presence of crowding agents and glycans [64,65].

Another useful tool used to evaluate protein self-assembly is the use of extrinsic probes that change its absorption upon binding to the protein aggregate. One of the most used ones is Congo red, which has been shown to bind with high sensitivity to amyloid fibrils [66]. After binding, a redshift from 490 to 540 nm is observed, as well as an increment of its light absorption [67]. This probe is used to follow the amyloid formation in solution at different conditions by a thermodynamic and kinetic approach [68,69], for example, to evaluate the aggregation of the superoxide dismutase 1 [70], a peptide fragment from the prion protein [71] and the β amyloid peptide [72].

### 2.3. Direct Methods: Analyzing Protein Aggregation in Solution

**Figure 4:**
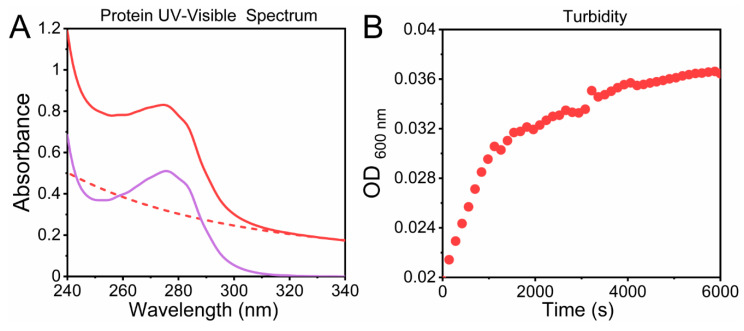
(**A**) Protein Spectrum (240–320 nm) where a high scattering contribution is observed (solid red line), the light scattering component (red dashed line) and the corrected spectrum (solid violet line). (**B**) Turbidity measurement of the aggregation process of C- terminal domain of TDP-43 protein in the presence of 300 mM NaCl. Here, the optical density at 600 nm was followed over time.

These methods directly detect particles in solution by the attenuation of the incident light due to the light scattered. One of the most used parameters is turbidity (τ), which is defined as the total light scattered in all directions by the following Equation (2) [51]:

(2) $$\tau =-\ln(I/I_{0})=-2.3\;OD$$

where OD (Optical Density) is defined as OD = A + S, where A is the absorbance and S the scattering of light. The presence of particles produces turbidity with sizes near 1/50–1/20 or above of the wavelength of incident light [51]. It is worthy of mentioning that an increment of the scattered light at shorter wavelengths might be observed (λ < 320 nm), affecting the region where the absorbance of the aromatic groups occurs. For this, it is recommended to correct the spectra due to scattering using an exponential decay curve (Figure 4A). To avoid the contribution of aromatic residues, turbidity is assessed in the non-absorbing region of the spectra (λ > 320 nm), where the principal component is the scattering of light. In this case, the relationship between turbidity and scattering is given by the following Equation (3) [73].

(3) $$\tau =-\ln(I/I_{0})=\left(16\pi /3\right).R\theta$$

where *I* is the direct transmission of light through the sample of fixed path length; frequently, these measurements are performed in the range of 350–600 nm.

This approach is used to make comparisons between the same samples under different conditions and to make a kinetic analysis of the aggregation of a protein upon time, as shown in Figure 4B for the C-terminal domain of TDP-43 protein. The advantages of this method are that it is easy to apply in highly aggregated systems, it has a low-cost, and it has high reproducibility. As relevant examples, we can mention actin polymerization kinetics, hemoglobin fibrillation [74] and liquid phase separation of TDP-43 C-terminal domain [75], alpha-synuclein aggregation and its effects on Tau protein [76], TTR amyloid aggregation in the presence of plasminogen [77], and the β-amyloid peptide [78]. From a pharmaceutical point of view, it is employed to evaluate the effect of stirring on the aggregation processes of an antibody, in the presence or absence of polysorbate 80 by using turbidity measurements (at 350 and 550 nm) [79].

Another parameter used is the aggregation index, which is frequently used to determine not only the presence of big aggregates in solution, but also helps to evaluate the existence of oligomers in solution, giving a first approximation of the aggregation state of the sample. The following Equation (4) defines this:

(4) $$AI=\frac{A_{350}}{\left(A_{280\;}-A_{350}\right)}\;*100$$

where $A_{280}$ and $A_{350}$ are the absorbances at 280 and 350 nm, respectively. When the aggregation index is below 3, the sample is a transparent solution. Between 3 and 30, some aggregation may exist, and above 30, the sample is heavily aggregated. This approach was used to evaluate the aggregation of the Voltage-dependent anion channels (VDACs) [80], gliadin protein [60] and the β-barrel transmembrane Ail from *Yersinia pestis* [81].

To sum up, UV-Visible spectroscopy is employed not only to quantify pure protein samples, but it is also a useful technique to quickly assess the protein and peptide aggregation process obtaining meaningful information about the molecular microenvironment of the aromatic residues of the system.

## 3. Fluorescence Spectroscopy Is a Versatile Tool for Evaluating Peptide and Protein Self-Assembly

### 3.1. Principles and General Considerations 

Fluorescence phenomena have become one of the most useful tools for the study of protein self-assembly and aggregation, with a wide variety of applications. Their great sensitivity, the non-destructiveness of the method with respect to the sample, and the possibility of carrying out real-time measurements enables the evaluation of the aggregation process from monomers to the formation of large assembled structures.

Fluorescence occurs when a fluorophore is excited from its ground state (S_0_) to a higher vibrational level of an excited state (S_2_ or S_1_) by the absorption of photons, the energy of which is equal to the energy difference between the two states. A fluorophore is a molecule that can absorb light of a specific wavelength and emit at a different wavelength after light excitation [82]. After the absorption process, the electrons that were promoted to a higher energetic level return to lower vibrational states by vibrational relaxation. This process is a non-radiative process in which collisions with the solvent produce a loss of energy. If the electron is in an S_2_, it could go the lowest energy S_1_ state by a process called internal conversion (IC), in which there is a jump between excited states due to their energetic proximity, and there is a loss of energy. After reaching the lowest vibrational state of S1, it could come back to the ground state through several processes, e.g., a non-radiative process due to interaction with solvent molecules or quenchers, fluorescence resonance energy transfer (FRET), or by a radiative process named fluorescence. In fluorescence, the excess of energy is emitted as a photon with a lower wavelength than the absorbed one.

**Figure 5:**
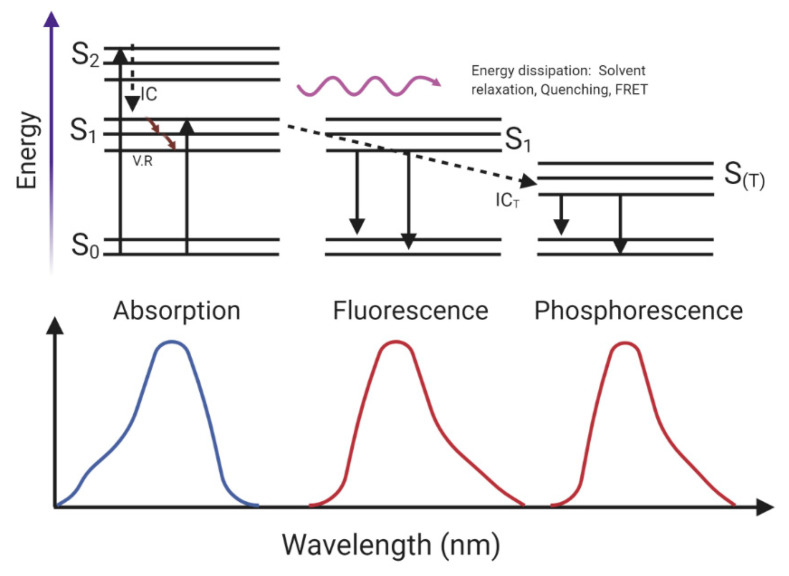
Simplified representation of the fluorescence and phosphorescence phenomena. The ground (S_0_) and excited states (S_1_ and S_2_) of a fluorophore are represented. The emission of fluorescence requires 10^−9^ s, while phosphorescence emission occurs from the triplet state (S_(T)_) in >10^−3^ s. For a detailed description, see text. This figure was created with Biorender.com.

The loss of energy occurs due to vibrational relaxation or collision with the solvent between the absorption and emission. In some exceptional cases, in the S_1_ lower state, a transition from a singlet to a triplet excited state (S_(T)_, spin conversion) could occur in a process called an intercross system (IC_T_). After that, electrons return to the ground state by non-radiative processes or by emitting light, which is called phosphorescence [83]. Figure 5 shows a schematic representation of this phenomenon. The fluorescence emission occurs at a longer wavelength than the absorption; the emission spectrum is similar to the mirror image of the absorption band of least frequency and, in pure samples, the shape and maximum of emission are the same independent of the excitation wavelength [84]. Due to the nature of fluorescence, its intensity is quantitatively dependent on the Lambert–Beer law, as described for UV-Vis.

**Figure 6:**
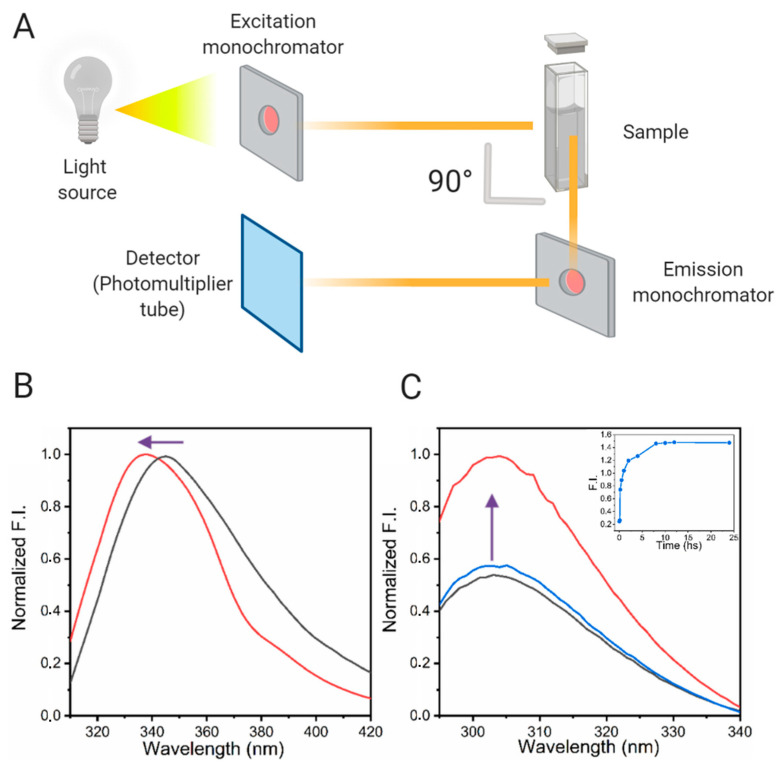
(**A**) Schematic representation of a Fluorometer instrument. (**B**) Tryptophan fluorescence spectra of a protein before (black) and after (red) the fibrillation process. Notice the blue-shift of the signal due to the change to a less polar environment. (**C**) Fluorescence spectra of a protein containing tyrosine (without any tryptophan), at 0 h (black), 1 h (blue) and 24 h (red) of the oligomerization process started. *Insert* a kinetic curve is presented. Notice the increment of the fluorescence intensity over time. This figure was created with BioRender.com.

When evaluating the aggregation of a protein of interest, it is essential to distinguish the different properties of the equipment used and the available experimental possibilities. The conventional spectrofluorometers are mainly composed of a Xenon arc lamp as a source of light, an excitation monochromator, the sample compartment, an emission monochromator, and the detector. It should be pointed out that the detector is placed at a 90° angle to the excitation beam in order to reduce the amount of exciting light in the observation direction (Figure 6A). In some equipment, a set of polarizers is set in between the sample and both monochromators, enabling anisotropy measurements. In other applications, like time-resolved fluorescence, the equipment used is slightly different. The source emits pulses of light to the sample, and the monochromator or filter selects the excitation wavelength. Then, polarizers are located before and after the sample. The detector is placed after this. Lastly, the detector is connected to a time-to-amplitude converter and to a multichannel analyzer. This configuration makes it possible to obtain a histogram of counts of fluorescence in the function of the time [84].

When performing and analyzing any fluorescence experiment, there are many technical aspects to take into account, which depend on both the sample type and the instrument [84]. In this sense, one important aspect is the absorption of the sample itself, because when there is a high local absorption or turbidity, an inner filter effect is observed. This effect consists of the attenuation of the excitation beam by the high absorbance of the sample, as a consequence, the only part of the sample that fluoresces is the one facing the excitation beam. Additionally, as the excitation and emission spectra overlap significantly, the light emitted in the center of the cuvette can be reabsorbed by the sample itself, producing a lowering of the signal and a distortion of the emission spectra. Notably, other types of experiments, such as time-resolved and anisotropy, are less affected by this effect. As a general recommendation, avoiding samples with a general absorption above 0.05 is desirable. If this is not possible, due to the low quantum of yield of the sample, it is recommended to use a cuvette with a shorter path length [82,84]. Another aspect to consider is the appearance of the Raman peak in the emission spectra. The Raman peak is the result of non-elastic scatter of water, and its position depends on the excitation wavelength. This phenomenon can be used for signal calibration in each experimental setup or to allow the comparison between spectra obtained in different instruments [85].

### 3.2. Intrinsic Fluorophore

Different approaches can be used to evaluate protein aggregation. We classify them based on the type of fluorophore used: intrinsic or extrinsic. The former takes advantage of the fluorescent properties of the aromatic amino acids tyrosine, tryptophan and phenylalanine. Tryptophan and tyrosine can be excited between 280 and 285 nm, depending on the polarity or pH of the solvent that could change their ionic state [86]. The maximal emission wavelength in water is 350 nm for tryptophan and 304 nm for tyrosine (Table 1).

Moreover, to avoid the emission of tyrosine, tryptophan can be selectively excited at 295 nm. Phenylalanine presents the shortest absorption and emission wavelengths (λex = 260 nm and λem = 282) and the lowest quantum yield. Tryptophan presents the highest quantum yield (0.14 over 0.13 of Tyrosine and 0.02 of Phenylalanine), which is the efficiency of photon emission as defined by the ratio of the number of photons emitted to the number of photons absorbed. Importantly, tryptophan is highly sensitive to its local environment emission spectra. In this sense, its emission spectrum is blue-shifted when the solvent polarity or solvent exposure is decreased, as happens when it is buried in a protein core. One of the most classic examples is tryptophan 48 from azurin [87], which is fully buried (λem = 308 nm) and presents redshifts to λem = 350 nm when the polarity of the environment increases, as occurs upon denaturation [88]. The magnitude of the shift depends not only on the water molecules surrounding the tryptophan, but also on the orientation of the indole ring.

In summary, tryptophan-containing proteins exhibit a fluorescence emission that is a linear combination of the emission of each tryptophan present in its sequence, and the spectra maximum could range from 308 to 350 nm, depending on the environment and orientation of the indole ring [87]. Because of this, many algorithms have been designed for the decomposition of the protein tryptophan emission spectra into its different components [89]. In the case of tyrosine, the change from a polar to a non-polar environment produces an increase in its fluorescence intensity. It is relevant to mention that when the tyrosine anion is formed, the maximum emission takes place at 340 nm (Figure 6) [90].

Interestingly, tryptophan fluorescence can be quenched by intrinsic quenchers present in the protein, such as amine, carboxylic acid, disulfide, and histidine groups, or external ones by the addition iodide and acrylamide, which are electron-rich species. Additionally, the time that a fluorophore spends in the excited state before emitting a photon and returning to the ground state, called a lifetime, is sensitive to the local environment, varying in the range between 1 and 6 ns [91]. Tyrosine fluorescence is useful for studying protein self-assembly when there is no tryptophan in the protein sequence, especially when combining it with acrylamide quenching [84,92]. Moreover, tyrosine is susceptible to quenching either by energy transfer to proximate tryptophan residues or through tyrosine/tyrosine homotransfer [82].

### 3.3. Extrinsic Fluorophore

Extrinsic fluorescence is obtained through the use of a variety of fluorescent probes that bind, in a non-covalent way, to hydrophobic patches, or they can be chemically attached to the molecule. These dyes are generally less fluorescent when they are in an aqueous environment, but when they bind to a hydrophobic pocket, which may be present in a protein aggregate, their quantum yield increases. In some of them, a blue-shift of the maximum of emission occurs [93]. The thermodynamic characterization of the binding of dyes to a protein aggregate is an essential tool for the evaluation of kinetic constants over different experimental conditions. One of the most widely used fluorophores is Thioflavin T (ThioT), which has a high selectivity for ordered aggregates such the ones that contain a large proportion of β-sheet structures [94].

As said above, fluorophores can also be attached to the protein of study by a covalent bond to the α-amino group of the *N*-terminus, the ε-amino group of lysine, or the thiol group of cysteine residues. The choice of the fluorophore and the methodology depends on the protein sequence and the implemented analytical technique. A critical issue to consider is the number of dyes bonded to the protein of interest, and also whether this could induce the aggregation of the protein. The number of fluorophore molecules attached to the protein can be determined using the molar extinction coefficients of both dye and protein [95]. To discount the probe inducing the aggregation of the protein, experiments could be carried out in the presence or absence of the probe.

Another exciting approach is FRET, which will be exemplified in the next section. In this case, the phenomenon relies on the distance-dependent transfer of the excitation energy from a fluorescent donor molecule to an acceptor molecule.

It is worthy of mentioning that there might be drawbacks in using intrinsic or extrinsic fluorophores. The main disadvantage of using the intrinsic ones is that their extinction coefficient and quantum yield are lower than the coefficients from the extrinsic dyes, and also that the process of quenching that could occur may reduce fluorescence intensity. In the case of extrinsic dyes, they could alter the protein structure, affecting the process under evaluation.

### 3.4. Assessment of Protein and Peptide Self-Assembly Processes by Fluorescence Spectroscopy: Methods and Selected Examples

A summary of the most widely used fluorescence spectroscopy set-ups is presented in Table 2. For detailed information about each of them, we suggest reading specialized books on this topic [82,96].

#### 3.4.1. Steady-State Fluorescence Methods

**Figure 7:**
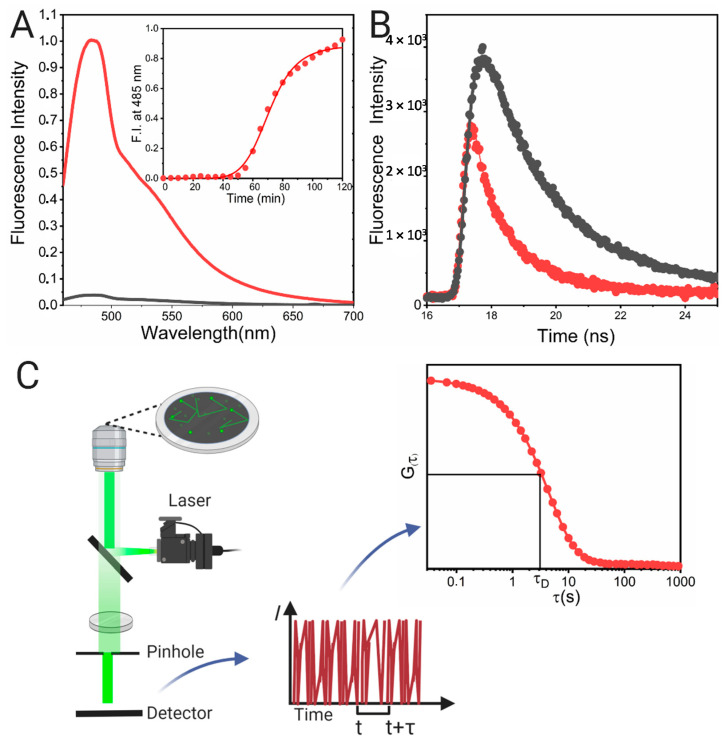
(**A**) Fluorescence spectra of Thioflavin T bound to BSA fibrils (red) in comparison to the probe without it (black). Inset: the kinetic of fibrils formation followed by fluorescence emission at 485 nm. (**B**) Lifetime curve of a Tyr following the aggregation process of a protein. Black and red curves stand for a higher and a lower protein concentration, respectively. (**C**) Fluorescence Correlation Spectroscopy (FCS). Left: schematic representation of a confocal microscope. In the upper image, we could see a detail of the observation volume and fluorescent particles diffusing. Right: the lower and the upper figures are generic examples of a correlation experiment. The lower graph shows fluorescence intensity versus time. The fluctuations are the product of the molecules transit in the confocal volume. The upper graph is the correlation curve obtained from the fluctuations. τ_D_ represents the half time of residence in the confocal volume. This figure was created with Biorender.com.

**Figure 8:**
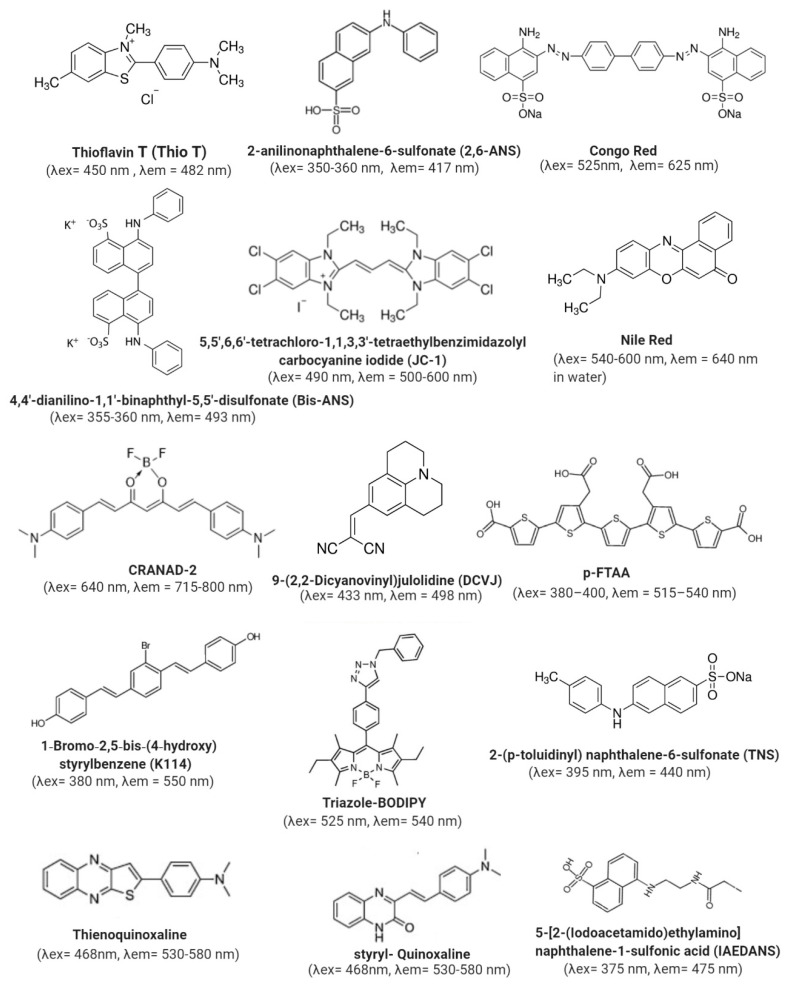
Structure of selected extrinsic fluorescent probes used to evaluate the self-assembly and aggregation of proteins and peptides. Image created with Biorender.com.

Steady-state fluorescence methods are the most widespread methods used to detect and characterize a variety of proteins and peptides that form self-assembly systems, such as the amyloid or colloidal like-systems (Figure 7A). One prominent example of amyloid systems is the Aβ-peptide implicated in Alzheimer’s disease, and its self-assembly into oligomers and fibrils. A variety of fluorescent probes are employed (Figure 8), such as Thioflavin T (ThioT) [97], Congo red derivatives such as K114 [98,99], pentameric thiophene derivatives (p-FTAA) [100], the curcumin derivatives CRANAD-1 (λex = 540 nm, λem = 640 nm), and −2 [101], thienoquinoxaline and styryl-quinoxaline derivatives [102] and boron-dipyrromethene (BODIPY)-based probes (λex from 500 to 650 nm, depending on the type) such as (BAP-1) (λex = 614 nm, λem = 648 nm) [103], among others. These probes interact with high sensitivity with Aβ-peptide fibrils, showing an increment in their fluorescence emission, and some of them, such as CRANAD probes, suffer a blue-shift of the maximum of the emission upon binding. Recently, a Triazole-BODIPY-based probe was shown to detect, in a time-dependent manner, the first stages of Aβ-peptide oligomerization before fibril formation [104]. Additionally, the same BODIPY derivative identified the presence of oligomers of the toxic p31–43 peptide related to celiac disease [49]. This probe has proved to be a helpful tool in the rational screening of drugs that reduce peptide aggregation before the onset of disease. Another protein involved in the pathogenic amyloid process is the alpha-synuclein protein, which occurs in Parkinson pathology. The structural properties of alpha-synuclein fibrils were deeply studied not only by probes such as ThioT [105], but also with *N*-arylaminonaphthalene sulfonate (NAS) derivatives such as 2-anilinonaphthalene-6-sulfonate (2,6-ANS) and 4,4′-dianilino-1,1′-binaphthyl-5,5′-disulfonate (bis-ANS), among others. It has been shown that NAS probes specifically bind to the fibrils with a Kd in the micromolar range [106]. Additionally, it was demonstrated that the cationic dye 5,5′,6,6′-tetrachloro-1,1,3,3′-tetraethylbenzimidazolyl carbocyanine iodide known as a JC-1 binds specifically to alpha-synuclein, and differentiates its different aggregated forms. JC-1 in solution presents two maxima of emission, one at 530 nm corresponds to the dye aggregates in water and another at 590 nm corresponding to the monomer [107]. Upon binding to the monomeric alpha-synuclein, the fluorescence intensity at 590 nm is increased; meanwhile, in the presence of fibrils the fluorescence emission at 530 nm shows an increment. Interestingly, in the presence of alpha-syunclein oligomers, a new fluorescent band at 560 nm is observed. Additionally, FRET between ThioT and this probe was detected, indicating that JC-1 is useful to monitor the different stages of alpha-synuclein self-assembly [107]. Another probe that has been demonstrated to detect alpha-synuclein oligomers is 4-(dicyanovinyl)-julolidine (DCVJ). This probe is a molecular rotor that self-quenches in solution due to intrinsic rotational relaxation, but upon binding to oligomers where the mobility is restricted, its fluorescence is increased [108].

On the other hand, proteins such as caseins and gliadins have been deeply studied using fluorescent probes, which have helped to evaluate and understand their colloidal nature. Caseins are of particular interest in the food industry, for example, because they form micelles which are implicated in calcium storage in milk, and due to their excellent properties as emulsifiers. Because of this, casein complexes formed with fluorescent probes that sense hydrophobic patches such as curcumin (λex = 420 nm, λem = 500 nm) [109], 2-(p-toluidinyl) naphthalene-6-sulfonate (TNS) [110] and Nile Red [111] were studied. These probes have helped in the design of possible nano-drug carriers. Gliadins are proteins present in wheat gluten and are related to celiac disease. Gliadin self-assembly in nanostructures has been studied by using the Nile Red probe. This probe has a particularity whereby the excitation state is different depending on the polarity of the medium. Because of this, the fluorescent emission depends on the excitation wavelength, and the molecules that are distributed in different micro-solvation domains can be selectively excited. This effect, where the emission maximum changes due to a change in the excitation, is called the “red excitation edge”, and it is observed in a variety of fluorescent probes [112], such as TNS [113] and aminobenzanthrone dyes (λex = 450–490 nm, λem = 560–640 nm, range depending on the dye) [114]. This phenomenon was used with Nile Red to analyze gliadin at pH 3.0 and 7.0. At acidic pH, a blue shift of the emission was observed (from λem = 640 to 608 nm) and two different microdomains were detected by exciting at 552 and 590 nm, due to a change in the emission spectra. Meanwhile, at pH 7.0, nearly no interaction was detected, and no change of the emission (λem = 640 nm) was observed in comparison with the buffer at either wavelength. This effect is characteristic of the presence of micelle-like structures at pH 3.0 and solid-like structures at pH 7.0 [60]. Additionally, Nile Red has also been used to perform a detailed study of the β-galactosidase aggregates [115]. To exemplify the steady-state fluorescence approach, an emission spectrum of BSA fibrils with ThioT is presented in Figure 7A (the inset corresponds to the kinetic binding curve).

Another steady-state tool for evaluating the formation of oligomers and higher-order structures is steady-state anisotropy (Table 2). In this case, the anisotropy value of the system increases when the correlation time of the studied protein varies as a consequence of self-association. For example, the Aβ-peptide labelled with a pyrene probe has been shown to sense the formation of oligomers by detecting a change in the anisotropy [116] (λex = 350 nm, λem = 460–500 nm (short live dimer or excimer) and 380–400 nm (monomer)). Additionally, a ruthenium polypyridyl complex (λex = 480 nm, λem = 605 nm) has been used to crosslink the formed Aβ-oligomers and follow the oligomerization process [117]. A similar approach labelling bovine serum albumin (BSA) with IAEDANS [118] was used to evaluate BSA aggregation. These examples showed the feasibility of using steady-state anisotropy as a routine method for protein self-assembly. However, it is worth noting that the fluorescent probe used has to be carefully selected depending on its lifetime, the size of the system to be explored, and the conditions in which the experiment would take place.

#### 3.4.2. Time-Resolved Methods

**Figure 9:**
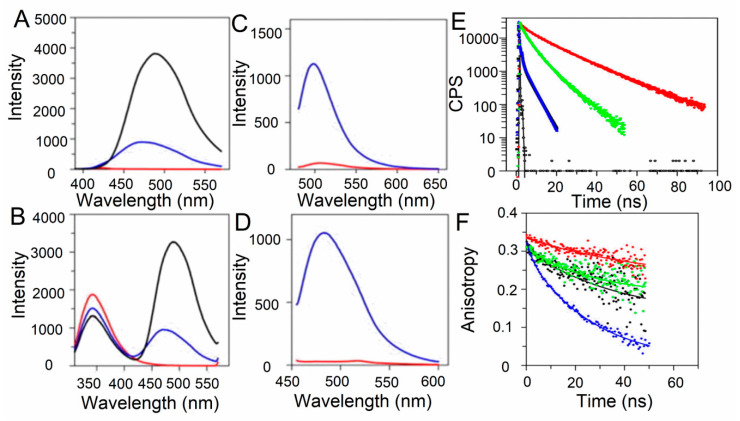
Fluorescence evaluation of the aggregation of TTR protein. (**A**) Fluorescence Spectra of ANS (blue line) and Bis-ANS (black line), after direct excitation, when bound to proto-fibrillar and spherical aggregates of TTR. The control without probes is presented in red. (**B**) Emission spectra excitation at 290 nm the proto-fibrillar and spherical aggregates of TTR in the presence of ANS (blue line) and Bis-ANS (black lines) and without probe (red line). (**C**) DCVJ spectra in the presence of native TTR (red line) and misfolded oligomers of TTR (blue line). (**D**) ThioT spectra in the presence of native TTR (red line) and misfolded oligomers of TTR (blue line). (**E**) Time-resolved fluorescence of the different probes bound to native TTR. ANS (red circles) or Bis-ANS (green triangles). DCVJ (blue triangles) and ThT (black squares) were bound to the misfolded aggregates of TTR after 1 h incubation. The ThT curve is hidden behind the DCVJ curve in the figure due to very similar decay profiles. The lamp response is indicated with open black circles. (**F**) ANS anisotropy decays of aggregates of TTR incubated for 24 h at different monomer concentrations: 2 μM (black), 4 μM (green), and 8 μM (red). As a comparison, the trace for native tetrameric TTR is shown in blue. This Figure was recreated and adapted from Lindgren et al. [119].

Fluorescence time-resolved methods, such as lifetime determination and anisotropy decay, are complementary techniques to steady-state fluorescence tools that help in the understanding of protein self-assembly at the molecular level. These techniques have been helpful in the evaluation of protein oligomerization and amyloid fibril formation. The characteristic decay curves obtained by time-resolved methods are shown in Figure 7A. One exciting example is the analysis performed by Lindgren et al. in the aggregation of Transthyretin (TTR) into protofibrils. The binding of this protein to fluorescent probes such as ANS, Bis-ANS (Figure 9A,B), and DCVJ (Figure 9C) and ThioT (Figure 9D) was evaluated by acquiring their emission spectra. Additionally, the tryptophan spectrum was obtained (Figure 9B). Based on a binding analysis, it was determined that these probes present a micromolar affinity to the protofibrils, but DCVJ has the higher one [119]. After that, time-resolved decay curves were obtained for each fluorophore in the presence of native (Figure 9E) and aggregated TTR. By fitting the fluorescence decay to an exponential curve, the lifetime (τ) of each dye in the presence of the protein fibrils was obtained. In the presence of TTR aggregates, an increment of τ was detected that could be explained by the formation of new hydrophobic patches or the reorganization of the existing ones due to a change in the protein structure and the polarity of the binding site. Additionally, the anisotropy decay showed an increment of the correlation time, in correspondence with the formation of higher-ordered structures. Examples of the curves obtained by these authors are presented in Figure 9F, where the anisotropy decay of TTR aggregates at different protein concentrations was evaluated [119]. The same approach using NAS probes was performed to sense changes upon alpha-synuclein aggregation [106]. Additionally, the study of the τ and correlation time of the pathogenic alpha-synuclein mutants, A30P and A53T, showed that they had distinct types of binding sites, which was in concordance with the difference in fibril morphology [120]. Additionally, the anisotropy decay profiles of fluorescein labelled-alpha synuclein have recently been used to study its liquid–liquid phase separation into droplets, an event that occurs before protein fibrillation [121].

It is worth noticing that the intrinsic fluorescence of the tryptophan and tyrosine residues is used for steady and time-resolved methods, as well. The study of oligomerization of the growth hormone in the presence of zinc [122], the fibrillation of the Aβ-peptide [123], alpha-synuclein [124,125], insulin [86] and Tau protein [126] are some remarkable examples to be taken into account. Figure 6B,C shows examples of changes in the steady-state fluorescence emission of tryptophan and tyrosine upon protein aggregation. Additionally, the di-tyrosine bond (λex = 320 nm, λem = 407 nm) is generally used as a valuable tool for cross-linking and characterizing oligomers and aggregates in different systems such as the 33-mer gliadin peptide [127], alpha-synuclein [125,128], β-amyloid peptide [129] and insulin [56].

#### 3.4.3. Fluorescence Correlation Spectroscopy (FCS)

Finally, FCS has been shown to be an invaluable tool for following protein self-association in complex systems (Table 2). In FCS, confocal microscopy is used to measure the fluorescent fluctuations that occur in a solution of a fluorescent labelled protein in confocal conditions. The rate of fluctuation is directly correlated with the rate of the molecule diffusion in the observation volume. This means that small molecules diffuse faster than bigger ones, which are detected as a rapid increase and decrease of the fluorescence intensity. Because of this behavior, the results are plotted as an autocorrelation function. By a least-squares fitting of this, the diffusion coefficient can be obtained. The inverse of the autocorrelation function at τ = 0 gives the average number of molecules being observed (G(0) = 1/N). FCS shares some of the principles of Dynamic Light Scattering (DLS), which is commonly employed to determine the size and shape of aggregates in general. The main difference is that in DLS, the intensity fluctuation of scattered light is measured. Both methods share the main principles of particle diffusion and data analysis. It is worthy of mentioning that when a protein aggregate, a wide distribution of different sizes is observed, and a continuous distribution fitting of the autocorrelation function has to be carried out rather than fitting by a discrete number of diffusion species [83]. In the same context, when the source of light used is X-rays, and the dispersion is collected at low angles, not only information about the size, but also the morphology of the particle could be obtained. This technique is commonly known as SAXS (small-angle X-ray scattering), and has been widely used to evaluate protein oligomers and higher-ordered structures in solution [130]. To get a better understanding of these complementary techniques, we recommend reading specialized literature [131,132,133,134].

For FCS approaches, the protein sample has to be labelled with a suitable fluorescent dye that has an adequate quantum of yield with low photo-bleaching at the working wavelengths (Figure 7C). This method was used to evaluate the oligomeric state of *Rubisco activase* protein by labelling with an Alexa 546 C5-maleimide (λex = 554 nm, λem = 570 nm). At different protein concentrations, the presence of dimers, tetramers, hexamers and higher-order aggregates was detected. The FCS technique has also been used to analyze the formation of the Aβ-peptide fibrils upon a time-lapse [135,136] and the early stages of oligomerization of alpha-synuclein [137]. Additionally, the assembly of viral capsid protein of the cowpea chlorotic mottle virus (CCMV) CP around short RNA molecules [138] shows the versatility of FCS for evaluating protein self-assembly under different conditions.

## 4. Circular Dichroism Makes It Possible to Follow Secondary and Tertiary Structure Changes Due to Self-Assembly

### 4.1. Main Principles and Structural Protein Features

Circular Dichroism (CD) is one of the most useful non-destructive techniques for measuring conformational changes in the secondary and the tertiary structure correlated with different processes, such as protein aggregation, thermal or chemical unfolding as well as binding interactions. This technique has the advantage of allowing the study of protein structural features in solution, mimicking physiological conditions, using a relatively low amount of protein, and the data are acquired in a short period. In this sense, complex processes such as the fibrillogenesis of collagen have been evaluated using CD spectroscopy [139]. This technique uses circularly polarized light in which the electric field rotates around the propagation axis at a constant magnitude. The type of polarized light could be left- and right-handed. Optical active compounds like proteins present differential absorption of those components producing an elliptically polarized light. As a result of this, the differential absorption is represented by the Lambert and Beer Law [140]:

$$\Delta A=A_{l}-A_{r}=\Delta \varepsilon \;cl$$

where ΔA is the absorption signal, $A_{l}$ and $A_{r}$ is the absorption in the left and right orientation of the circularized light, respectively, and Δε is the difference between the left- and right-handed molar extinction coefficients; c is the concentration and l the length of the optical path.

However, CD is generally reported in ellipticity θ:

$$\theta ={tan}^{-1}(\frac{b}{a}),$$

where b is the minor and a is the major axes of the resulting ellipse.

There is a numerical relation between ΔA and θ (in degrees):

θ=33 ΔA

The plot of ΔA, θ or Δε against the change of wavelength is the circular dichroism spectra. In the case of proteins, the CD spectra are normalized by the following equation:

$$\left[\theta \right]MRW=\frac{\theta \;M}{c\;l\;n_{r-1}}$$

where [θ]MRW is the mean molar residue ellipticity in degree cm^2^/mol, M is the molecular weight, c is the concentration in g/cm^3^, l is the path length in cm, and $n_{r-1}$ is the number of residues of the protein minus 1.

**Figure 10:**
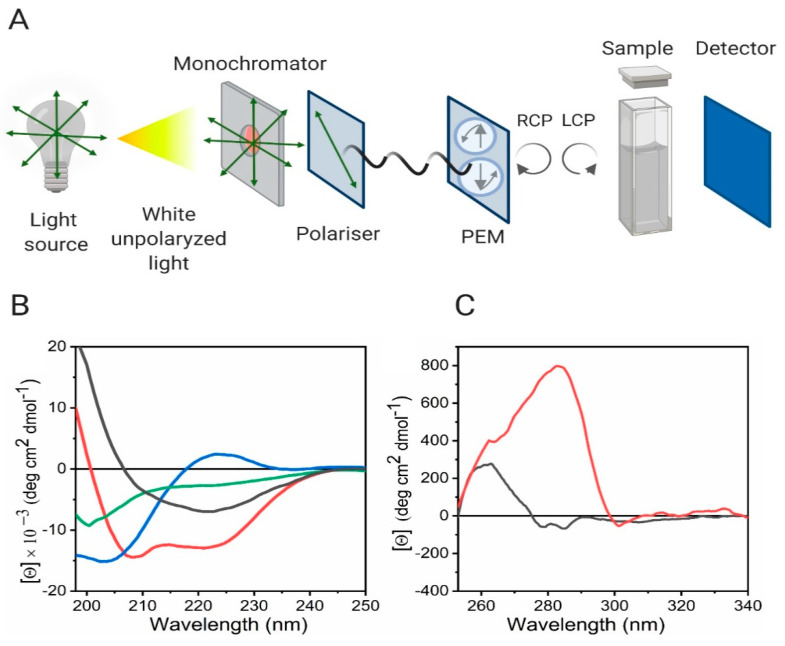
Circular Dichroism as a tool for the study of protein secondary and tertiary structure. (**A**) Schematic representation of the Circular Dichroism instrument configuration. (**B**) Representative Far-UV CD spectra of the characteristic secondary structure motifs detected in proteins and peptides: random-coil (green), α-helix (red), β-sheet (black) and Polyproline II (blue). (**C**) Near-UV–CD spectra of two different proteins, one containing tryptophan and tyrosine (red) and the other only tyrosine (black). This image was created with Biorender.com.

There are two central regions to study protein absorption by CD, the Far-UV and the Near-UV regions. In the first, the amide bonds absorb, whereas in the second, the absorption of lateral chains of aromatic residues and cysteine occurs. In the Far-UV region, two electronic transitions are observed, n→π ∗, which is observed at around 220 nm as a negative band, and which is sensitive to hydrogen bond formation, and π→π ∗, which is observed as a positive band at around 190 nm and a negative band at around 210 nm. These bands give an idea of the secondary structure composition of the studied protein or peptide. For example, the α-helix presents two negative bands at 208 nm and 222 nm and a positive one near 193 nm. For the β structure, a broad negative band around 215–225 nm and a significant positive band at 195 nm are observed. The CD signal of the latter structure is less intense than the α-helix signal and includes the different types of arrangements of β-sheets and turns [50,141]. As mentioned previously, the PPII is increasingly recognized as an important element in peptide and protein function. CD is a suitable method to study this conformation due to its distinct signatures which are an intense negative band at 206 nm and a weak positive band at 225 nm [44]. In the case of disordered structures, a unique negative band near 200 nm is observed. Recently, the CD spectrum of a synthetic designed α-sheet peptide has been described as featureless, due to a near equal absorbance of both left and right-handed polarized light, except for a negative signal around 195–200 nm, coming from residues in turns and terminal residues [142]. In Figure 10B, the most representative spectra of the secondary structure motifs detected in proteins are shown. Considering the structural features described, a change of CD spectra depending on time or/and varying the specimen concentration is a characteristic feature of a self-assembly process in solution.

In the Near-UV region (350–250 nm), the absorption of tryptophan, tyrosine, and phenylalanine can be used, but other chromophores could also be detected in this region. The aromatic residues do not have an intrinsic CD signal due to the planarity of their rings; however, when they are located in a chiral environment, they could lead to absorb the left- and right-handed circular polarized light. The main drawback to using the Near-UV-region for analysis is the high amount of protein/peptide required due to the low number of chromophores in the sample, and also that only a spectral comparison is possible. For these reasons, it is seldom used to perform a detailed structural analysis of protein (Figure 10C) [140].

### 4.2. Experimental Considerations of CD Spectroscopy

From an experimental perspective, the equipment used in CD is similar to the classical UV-Vis spectrometer, but in CD between the sample and the monochromator, there is a light polarizer and a photo-elastic modulator. The latter is one of the most widely used dispositive for producing left- and right-handed circular light. The light that emerges from the sample goes to the detector, which is connected to an amplifier and a modulator, and there the data is processed (Figure 10A). Due to the interference of oxygen_,_ the measurements take place in a nitrogen atmosphere.

There are some critical parameters to consider before performing any CD spectra: the selection of the buffer, the cuvette, and the concentration of the protein used. The selected buffer needs to be compatible with the protein, and it is suggested that it absorbs as little as possible in the range where the measurement will be performed. Generally, inorganic buffers, such as phosphate with low salt concentrations, especially low chloride, which is strongly absorbent, are the best options for CD. Kelly et al. [143] presented a list of some of the most widely used buffers and their concentrations for CD. In Far-UV, rectangular cuvettes with a pathway from 1 to 0.1 mm are generally employed. The need for a smaller path-length, 1 mm, is due to the high content of peptide bonds present on the sample. Additionally, when the sample presents a high concentration or high aggregation propensity, a saturation of the signal is observed.

In this case, sample dilution is needed or, when it is not possible, a cuvette of a smaller path-length, such as 0.1 mm, is preferred. For example, when protein aggregation is studied in a concentration-dependent manner, the spectra must be obtained using the correct cuvettes, as this is needed to avoid signal saturation. In this case, after the analysis of the spectra, a hypochromic displacement of the negative band is typically observed due to the higher ordered species in solution. For Near-UV, the cuvettes used usually present 5 to 10 mm path-lengths. As mentioned, the reason for using this type of cuvette is the low quantity of chromophores absorbing circularly polarized light in this region. The protein and peptide concentration have to be selected and determined with accuracy. This is necessary because most of the protein spectra are expressed in terms of mean molar residue ellipticity. The most used method for protein quantification, in a pure protein system, is the absorbance at 280 nm, as mentioned for UV-Visible section [140,143].

In terms of the data processing, the Far-UV CD spectrum obtained is a linear combination of the signal of each secondary structural element presents in the system. For a detailed analysis of the precise structural composition of the sample by Far-UV CD, many algorithms were developed. DICHROWEB, is one of the most used websites that combines some of the most popular algorithms as SELCON3, CONTIN and CDSSTR, among others to fit the experimental data [144]. Recently, BESTSEL website has been developed which provides a novel useful algorithm to predict not only the secondary content but also to define the type of β- structure as parallel, anti-parallel and turns, which is particularly helpful in the study of protein aggregation [145].

It should be pointed out that for a detailed study of β-sheet, IR spectroscopy is preferred because it is highly sensitive to evaluate and discriminate in between different β structures. In the case of protein samples, the backbone and some lateral chains present typical IR absorption signals, being the Amide I region the most sensitive to secondary structure changes, primarily upon aggregation [146,147]. Due to the interference of water signals in the spectra acquisition, the best way to evaluate proteins by IR spectroscopy is by performing the measurements with the Attenuated Total Reflectance technique. In this case, the sample needs to be deposited as a hydrated film in a germanium crystal for the spectra acquisition. Its advantages are that this technique uses a low amount of sample, the knowledge of the protein concentration is not required, and it is not disturbed by the presence of precipitates. This method has been mainly used to evaluate the secondary structure of protein oligomers and aggregates; especially in the case of amyloid fibrils [148,149,150,151]. Recently, the use of ATR-IR coupled with AFM, was reported as a method to simultaneously determine secondary structure and morphology of the aggregates [151].

### 4.3. Uses of Far-UV CD Spectroscopy on the Study of Self-Assembled Systems

#### 4.3.1. Analysis of Amyloidogenic Proteins

CD is widely used to evaluate the formation of amyloid fibrils at different experimental conditions such as time, changes in pH, and presence of different molecules, among others. The Aβ-peptide [104], the Ac-Phe-Phe-Cys-NH_2_ (Ac-FFC-NH_2_) amyloid peptide model [61], and proteins such as alpha-synuclein [152] and β2-microglobulin [153] have been shown to change their conformation from random coil to β-sheet structure over time during the aggregation process, which is compatible with the formation of fibrils. Other systems, such as the rationally designed DN1 peptide, have been shown to self-aggregate into fibrils in a concentration-dependent manner [154]. The β2-microglobulin protein presents an equilibrium in between amorphous and amyloid fibrils depending on the temperature and the salt concentration. In both types of aggregates, there is a conformational change towards a β-sheet structure [153]. The fibrils of this protein also present a high pH dependence with respect to stability [155]. Proteins such as the Hen-egg lysozyme [156], the regulatory ATPase variant A (RavA) from *E.coli* [157], TTR [158], serum amyloid A [159], insulin [160], and glucagon [161], among others, have been shown to aggregate at acidic conditions (pH = 2–5), where the transition to a β-sheet structure upon oligomerization and amyloid aggregation was observed. However, other peptides and proteins, such as the designed EASZ model peptide [162], Ac-Phe-Phe-Cys-NH_2_ (Ac-FFC-NH_2_) [61] amyloid peptide model, and some peptides from human Pbx-regulating protein-1, have been shown to aggregate to β-sheet structure at basic pHs [163].

**Figure 11:**
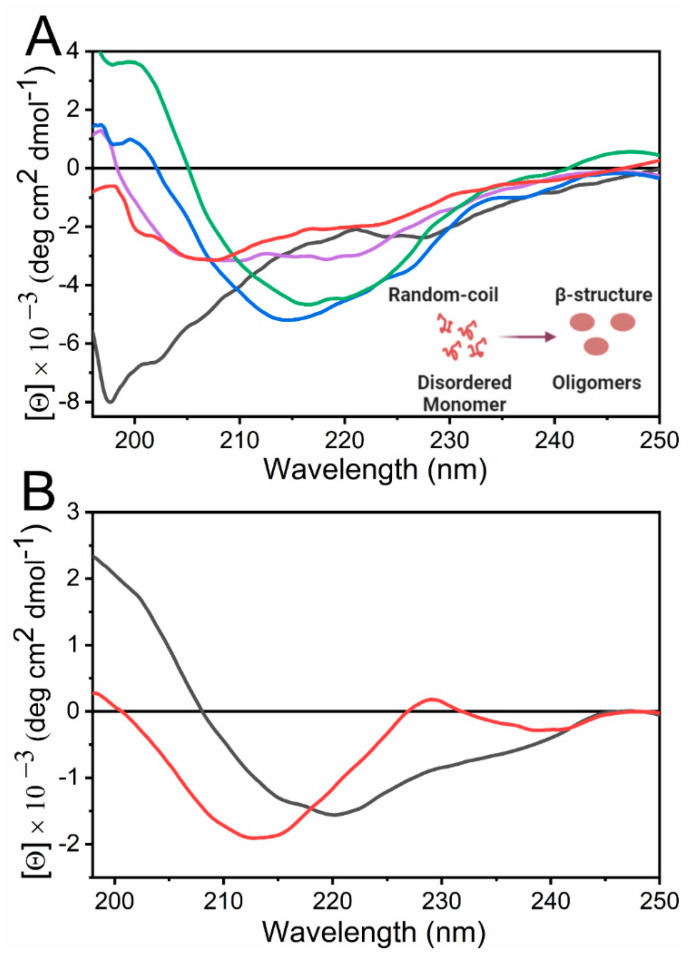
Circular Dichroism as a tool for the study of protein self-association. (**A**) Experimental spectral analysis of the oligomerization of the αβ-amyloid peptide overtime under physiologically relevant conditions. In 0 (black), 0.5 (red), 1 (violet) 3 (blue) and 7 h (green). This image was extracted and re-created from Tonali et al. [104]. (**B**) Spectra showing the structural transformation of the 33-mer gliadin peptide at 613 µM depending the temperature, at −5 °C (red) corresponds to PPII structure and at 37 °C (black) to the β-sheet signature. Image re-created from Herrera et al. [48].

In the case of proteins such as Tau [164], Gelsolin [165] and apolipoprotein A-I [166], a similar aggregation and structural behavior were observed upon exposure to molecules such as heparin. In those cases, the proteins adopt a typical β-structure in the presence of this molecule, suggesting that the extracellular environment influences the tissue-selective deposition of amyloids. Figure 11A represents the conformational change discussed here, showing the conversion of β-amyloid peptide from random-coil to a β-sheet structure that corresponds to the formation of oligomers. It is worthy of mention that CD has also been used to study the effect of a variety of molecules that could reduce or inhibit the conversion to native proteins and peptides to amyloids aggregates. For example, the effects of vitamin B12 [167], hydralazine [168] and carbenoxolone [169] were studied on the aggregation of β-amyloid peptide and curcumin [170] on lysozyme amyloid fibril formation, among others.

#### 4.3.2. Evaluation of Non-Amyloid Systems

Additionally, the aggregation of peptides and proteins into non-amyloid fibrils has also been extensively studied by CD. The 33-mer and the p31–43 gliadin peptides, which are related to celiac disease, have been shown to self-organize into oligomers and non-amyloid fibrils. The 33-mer peptide has been shown to form higher-ordered structures when the concentration is increased from 46 to 613 µM. By the use of CD spectroscopy, a conformational change from an unordered structure to a more folded one due to self-assembly was detected from the low to the high concentration. This change was accompanied by a hypochromic displacement of the negative band, which is indicative of light scattering as a consequence of the presence of large particles. Additionally, this peptide has been shown at 613 µM to present a clear dependence with the temperature. At this concentration, a redshift of the negative band from 210 nm at 5 °C to 220 nm was detected at 37 °C, indicating the formation of a β-sheet structure, which is shown in Figure 11B [48]. The p31–43 peptide has been shown to aggregate into oligomers, but in this case, the peptide presents a stable PPII structure. As the concentration increases, a hypochromic displacement of the negative is detected [49]. In the case of proteins, the β-lactoglobulin, which is an α, β structured protein, have been shown to self-aggregate in a concentration-dependent manner which was detected by CD. Therein, it was also observed a hypochromic displacement of the CD signal accompanied by the increment of the β-sheet structure [171]. Additionally, the formation of fibrils by this protein with a stabilization of the α-helix structure and an increment of the β-sheet signal was observed upon continued heating at 80 °C [172]. Superoxide Dismutase 1 has been shown to form oligomers and other non-amyloid aggregates in the presence of calcium, which correlates with an increment of the CD signal at 218 nm compatible with the appearance of a β-sheet structure [173]. Additionally, the self-assembly of the surfactant protein A, which is an oligomeric protein present in the alveolus acting against pathogens, has been studied by CD. This protein showed a decrease in the CD negative band (with a minimum at 207 nm) due to increasing calcium and sodium concentrations, indicating an aggregation process [174]. In the case of monoclonal antibodies, the stability and aggregation of different formulations at different pHs, temperatures and incubation times were followed by CD [175].

Another widely applied method in the evaluation of the secondary structure of the protein is to follow the CD signal at a fixed characteristic wavelength during the time, which is known to change through the aggregation process. In globular proteins, the range 218–222 nm is generally selected because of their content of α-helix and β-structures, which change upon aggregation typically because of increment of light scatter. This approach was used to monitor the aggregation of the wild type and the mutants W66Y, Y16W of the asparaginase-2 protein from *E. coli*, the complex of human apolipoprotein C-1 (apoC-1, Mw = 6 kD), and dimyristoyl phosphatidylcholine (DMPC) and monoclonal antibodies during a thermal unfolding [175,176].

#### 4.3.3. The Use of CD to Assess Conformational Equilibriums during Self-Assembly by Changes on the Microenvironment

It is worth noticing that for a better understanding of protein structure and how changes in the microenvironment of the protein affect its folding, different co-solvents are needed. Two of the most widely used compounds are trifluoroethanol (TFE) and sodium dodecyl sulfate (SDS) [177]. TFE affects the polarity of the solvent, making it more apolar and less basic. This change produces a micro-solvation effect that mimics long-distance interactions with tertiary structure. As TFE reduces the formation of hydrogen bonds, the interactions established with the solvent are reduced, and an increase of the intra-molecular ones is observed, which could lead to an increase in the formation of secondary structures, such as α-helix and β-sheet [178,179,180]. In the case of SDS, this surfactant provides the negative charges that interact with the positive residues on the protein, and the hydrophobic region makes contact counterpart by repelling the water, creating a hydrophobic effect that perturbs solvent–protein interaction. It has been shown that when its concentration is below its critical micelle concentration (CMC), the formation of β-strands is favored, but when it is higher than the CMC, α-helix is stabilized [177]. Due to the effects that these molecules produce on the secondary structure, they have been used to evaluate protein self-assembly. In the first studies, both molecules were used to assess by CD changes in the folding and oligomeric state of selected α-helix and β-strand peptides. The authors showed that, independently of the primary structure, a marked α-helical structure in TFE and β-strand in non-micellar SDS appeared [177]. In the study of the aggregation of Aβ-peptide (1–40), it was presented that TFE induces an intermediate helix which accelerates their fibrillogenesis in a concentration-dependent manner [181]. The same kind of structural behavior was observed in the case of the wild-type alpha-synuclein and its pathogenic mutants, showing that before the β-strand amyloid formation, an α-helical intermediate occurs [182]. Additionally, a detailed study in the influence of TFE concentration in insulin amyloid fibrillation was performed in the presence or absence of ultrasonication. This evaluation helped to propose a mechanism of fibrillation in which the metastability and supersaturation of the system have a critical role [183].

In some cases, the protein sequence restricts the folding, and in the presence of TFE, other structures are induced. This effect is observed in the case of proline-rich peptides, given that proline is s a helix breaker. For example, in the case of the 33-mer and p31–43 gliadin peptides rich in prolines, both have a PPII structure, which in the presence of increasing concentrations of TFE converts to a β-sheet-like one [48,49]. This equilibrium occurs due to the propensity of PPII to interconvert into β turns and β-strands [44]. TFE has also been used to study the aggregation of human muscle acylphosphatase into fibrillar and non-fibrillar aggregates. After destabilizing the protein by heat or urea, an incubation with different concentrations of TFE was performed, showing the presence of at least three different structural intermediates, one with a high percentage of α-helical structure, other with β-sheet and the last one mostly unstructured. However, all of those structures progressed to the formation of an amyloid structure [184]. SDS has been used to evaluate the aggregation tendency of the non-amyloid β-component (NAC) 71–82 amino acid stretches of α-synuclein. This region was shown to present a structural transition from random coil to β-sheet when SDS was below its CMC; however, when increasing the SDS concentration over the CMC, an α-helix structure is stabilized. It was pointed out that SDS induced the formation of amyloid fibrils in a variety of proteins when they are in partially folded states, including gelatin, concanavalin A, and hemoglobin, suggesting that SDS could be used as a tool to study the amyloid aggregation of proteins [185].

### 4.4. Near-UV CD Analysis of Protein Aggregation

Near-UV CD spectra are usually collected in the absorption range of cysteine and aromatic residues. This analysis has helped in the identification and evaluation of protein tertiary structure, especially to compare protein variants, as it was employed in frataxin [186,187], the intestinal fatty acid-binding protein [188], alpha-synuclein [189], and oligomeric proteins such as the hypoxanthine phosphoribosyltransferase [190]. In this way, the perturbation in the aromatic side chains due to aggregation could be reflected in this region. For instance, proteins from the crystalline, which are present in the eye lens, were studied after UV-radiation exposure, showing that these proteins change their tertiary structure upon UV exposure and form oligomers with a consequence in the understanding of cataracts [191]. Additionally, the aggregation of the Josephin domain of ataxin 3, a protein which polyglutamine expansion induces the formation of aggregates, was studied in the presence of different concentrations of TFE, showing a marked change in the tertiary structure of the protein, accompanied with changes in the aggregation kinetics [192]. In the case of insulin, a detailed study using Near-UV CD was carried out to obtain information on its tertiary structure behavior at high concentrations of NaCl with or without chaotropic agents as urea. For this, phase diagrams were made, plotting the molar ellipticity at 253 and 273 nm. These diagrams showed that NaCl by itself was able to induce structural changes in insulin, and in combination with urea, a further modification on this hormone structure was induced [193]. All these examples suggest that Near-UV CD could be a complementary qualitative tool to follow protein self-organization in solution.

## 5. Some Considerations in Order to Avoid Protein Aggregation

Apart from aggregation and self-assembly studies, it might be of interest to study the monomeric form of the different proteins with prone aggregation or self-assembly tendencies. To perform this kind of study, different strategies could be applied, depending on the protein under investigation. The agents that may be used for these purposes are extensive, with chaotropes, aminoacids, detergents, sugars and polyhydric alcohols, and polymers being among the most used. The mechanisms by which these agents avoid aggregation are diverse, and in some cases are not entirely understood. Some of these agents may decrease the rate of protein association and dissociation by being kept out from the protein–protein encounter surface, with the condition of not interacting with the protein monomer [194]. For instance, in the works on the ISD11 protein (part of the Fe-S cluster mitochondrial supercomplex in eukaryotes), SDS (0.45 mM) and DDM (1 mM) were used to help the protein refolding and prevent aggregation [195]. Another interesting example to avoid aggregation is the addition of glucose (up to 5%) in the protein buffer (apart from the buffer agent, salts, and any other co-solvents) of the UDK-c protein from *D. melanogaster* [196]. In a more biotechnological approach, there are many examples of *E. coli* acting as a chaperone of co-expression to obtain soluble proteins [197]. It is important to mention that, in general, the combination of co-solvents is useful to avoid aggregation [198]. In addition to the decision as to the type and amount of co-solvent to be used, it is relevant to take into consideration the kind of experiment that we are attempting to perform afterwards, i.e., we should avoid using high salts and glycerol concentrations to perform functional studies including DNA and DNA binding proteins [196].

## 6. Conclusions

In this review, we described the principles, uses, and several meaningful examples of UV-Vis Absorption Spectroscopy, Fluorescence Methods, and Circular Dichroism employed in the area of protein and peptide self-assembly. The main advantages of these methods are their availability in most research laboratories, their versatility and their quick data analysis.

As we mentioned before, no isolated method of analysis could lead to a complete and exhaustive study of any of these phenomena. The spectroscopic methods described here, in combination with other spectroscopic and microscopic techniques, have already helped in the elucidation of the self-assembly process of many proteins or peptide systems. Finally, the spectroscopic techniques described here remain the most widely used for performing an initial integral analysis of protein and peptide self-assembly processes, because of their availability and easy analysis.

## Figures and Tables

**Table 1:** Absorbance and fluorescence of aromatic amino acids and cysteine in water. In the lower part, it is presented how to calculate the extinction coefficient of a protein, Φ is the quantum yield.

	Absorbance			Fluorescence	
Compound	λ max (nm)	ɛ max (M^−1^ cm^−1^)	Ɛ280 (M^−^^1^ cm^−^^1^)	λ max (nm)	Φi
Tryptophan	280	5600	5500	355	0.14
Tyrosine	275	1400	1490	204	0.13
Phenylalanine	258	200	200	282	0.02
Cysteine			125		

ε_280_ (prot) = Number (Tyr) × ε_280_ (tyr) + Number (trp) × ε_280_ (tyr) + Number (Cysteine) × ε_280_ (Cysteine) [50].

**Table 2:** Description of the most widely used techniques in fluorescence spectroscopy to characterize protein self-assembly in solution.

Method	Principle	Application
Steady-state fluorescence	Generally, the emission spectrum of the sample is acquired by exciting the sample at a specific wavelength. The emission maximum, the shape of the spectra and intensity could be analyzed to obtain information about the local environment in the protein.	1. Monitor protein unfolding and aggregation using intrinsic and extrinsic fluorophores. 2. Evaluate the effect of tyrosine or tryptophan quenchers to evaluate solvent exposure
Steady-state anisotropy	Consists of the illumination of the sample with polarizers parallel to the Z axis. The fluorescence of the sample is collected firstly with the emission polarizer oriented (1) parallel to the excitation polarizer (I||) and (2) perpendicular to the excitation polarizer (I⊥). The following equation (5) describes how anisotropy (r) is obtained: $$r=\frac{I||\;-\;I⊥}{I||+2I⊥}$$ There is a direct relation with the correlation time $$r=r_{0}/\left(1+\frac{\tau }{\theta }\right)$$ where: $r_{0}$ is the anisotropy at time 0, τ is the lifetime and θ is the correlation time.	1. Anisotropy measures the rotational displacement of a molecule during the lifetime of the excited state of the fluorophore. 2. It is sensitive to the size of the molecule and local mobility. In the first case, the value increases when increases the size of the particle
Time-resolved spectroscopy	The sample is excited with a light pulse of a specific wavelength, and the decay of the signal emission is detected over time. By this approach, the lifetime of the fluorophore (τ), is determined, which is the time that the emitted intensity decays to 1/e of the original value. The equation is: $$I_{t}=\alpha e^{-t/\tau }$$ where $I_{t}$ is the intensity at time t, α is a normalization term, and τ is the lifetime. The same approach but using polarizers for the excitation and emission as it was described above could let study the dynamic of the fluorophore known as an anisotropy decay.	1. It determines the origins of the changes detected by steady-state anisotropy. 2. It shows the presence of different species in the sample, for example, different oligomeric species that binds to a fluorophore.
Fluorescence correlation Spectroscopy	The sample is settled in a coverlid, and it is illuminated by the laser source of a confocal microscope. The molecule of interest must be in a low concentration to have a small number of particles. In this approach, the fluctuations of the fluorescence due to diffusion are recorded. The results obtained are reported as an autocorrelation function, described as below $$G\left(\tau \right)=\frac{\langle F_{\left(t\right)}F_{\left(t+\tau \right)}\rangle }{{\langle F\rangle }^{2}}$$ where $F_{\left(t\right)}$ is defined the intensity at time t, F(t), and the intensity at a later time and F(t + τ) is the Fluorescence at a time point t + τ, averaged over a large number of measurement. For protein oligomerization, there are two approaches photon counting histogram (PCH) and the fluorescence-intensity distribution analysis (FIDA). In both, the essential parameter is the inherent molecular brightness (B) of the fluorophore, which is the counts per second per molecule (CPSM). The idea is to measure the signal from a standard fluorophore, and then, with the same instrumentation settings, the sample is analyzed. The brightness parameter is proportional to the number of molecules of fluorophores under observation.	1. It determines the diffusion coefficients and hence the size distribution of the aggregates. (the equivalent of dynamic light scattering) 2. In PCH and FIDA the oligomerization state of the sample is obtained
